# Enhanced Quarter Spherical Acoustic Energy Harvester Based on Dual Helmholtz Resonators

**DOI:** 10.3390/s20247275

**Published:** 2020-12-18

**Authors:** Xincun Ji, Lei Yang, Zhicheng Xue, Licheng Deng, Debo Wang

**Affiliations:** College of Electronic and Optical Engineering & College of Microelectronics, Nanjing University of Posts and Telecommunication, Nanjing 210023, China; jxc@njupt.edu.cn (X.J.); lyang202020@126.com (L.Y.); xzc10101@163.com (Z.X.); dlc@njupt.edu.cn (L.D.)

**Keywords:** AEH, Helmholtz resonators, frequency band, multi-directional collection

## Abstract

An enhanced quarter-spherical acoustic energy harvester (AEH) with dual Helmholtz resonators was designed in this work. Compared with the previous research, this AEH can harvest multi-directional acoustic energy, has a widened resonance frequency band, and has an improved energy conversion efficiency. When the length of resonator’s neck is changed, the acoustic resonant frequency of the two resonators is different. The theoretical models of output voltage and output power were studied, and the relationship of output performance with frequency was obtained. The results showed that this AEH can operate efficiently in a frequency band of about 470 Hz. Its output voltage was found to be about 28 mV, and its output power was found to be about 0.05 μW. The power density of this AEH was found to be about 12.7 µW/cm^2^. Therefore, this AEH could be widely used in implantable medical devices such as implantable cardiac pacemakers, cochlear implants, and retinal prosthesis.

## 1. Introduction

In the past few decades, more and more attention has been paid to collect energy from the environment [1]. Compared with conventional energy supply devices such as batteries, an energy harvester has the advantages of a longer lifetime, a smaller size, and better reliability. External energy sources mainly include vibration energy [2], solar energy [3], magnetic energy [4], and acoustic energy [5,6]. Among them, acoustic energy has become a hot spot because it is widely distributed in the environment. It is also a renewable and clean power source, especially in implanted and the environmental monitoring devices. However, acoustic energy harvesters (AEHs) have a low energy collection efficiency based on a piezoelectric cantilever [7] or a fixed beam [8], and this makes it difficult to apply traditional AEHs.

In order to improve output performance, three main kinds of AEH based on resonator structures have been recently widely researched: Helmholtz resonators, quarter-wave resonators, and acoustic crystals.

(1) Helmholtz resonators are based on the Helmholtz effect, which can periodically amplify an incident acoustic wave [9,10,11]. Farid Khan et al. proposed an AEH with a tapered Helmholtz resonator, and they improved the output performance by changing the cavity structure. It could receive an output power of 90.6 L μW with an excitation of 130 dB [12]. Xiao Peng et al. designed a coupled AEH, and the output power could be increased by up to 16 times compared with a single resonator [13].
(2) Quarter-wave resonators are optimized based on Helmholtz resonators, and the length of the cavity is equal to a quarter of a wavelength [14]. It can harvest acoustic energy with a smaller volume, which saves space in a particular structure. Bin Li et al. designed an AEH with a quarter-wavelength straight-tube acoustic resonator, and several piezoelectric cantilever beams were placed inside the resonator. The largest sound pressure amplification ratio was about 59.1 at an incident sound of 100 dB [15].
(3) Acoustic crystals use a point defect as a resonator [16]. A point defect in a structure is caused by the removal of a rod from the perfect sonic crystal. It can significantly improve output performance. Liang-Yu Wu et al. designed and put an piezoelectric material in a point defect; their results showed that the acoustic power was about 24.4 times larger than that without a sonic crystal [17].

Though these AEHs based on resonator structures have improved output power, some shortcomings still exist. On the one hand, energy harvesting is single-direction, and most of the environmental acoustic energy from other directions is wasted. On the other hand, the resonant frequency band is narrow, and the energy conversion efficiency is low. In recent years, some new structures have been presented. Hong-Fa Zhao et al. investigated a novel dual-tube Helmholtz resonator-based triboelectric nano-generator. Their experimental results showed that the power density of the fabricated device was 1.82 WPa^−1^ m^−2^, which was higher than the best results from the literature by 20% [18]. Pejman Eghbalia et al. designed an AEH with an axenic latticed resonator backed by an acoustic rectangular tube. It could arrive at a large magnification factor of around 10.5 for a 100 dB sound pressure level at resonance [19]. Iftikhar Ahmad developed an AEH that consisted of two Helmholtz cavities and a commercially available piezo element. The experimental results indicated a maximum power density of 32.7 μW/cm^3^ at 130 dB [20].

However, none of these structures could solve the problems of insufficient external excitation, single energy collection direction, and narrow resonance frequency band at the same time. In this work, an enhanced quarter-spherical AEH with dual Helmholtz resonators is proposed to achieve multi-direction energy harvesting, a wide frequency band, and a high energy conversion efficiency. Since the opening directions of the two coupled resonators were different, this system could harvest energy from different directions. Since the acoustic resonant frequency was related to the device’s size, a wider resonant frequency band could be obtained by optimizing the size of one neck and thereby improving the energy conversion efficiency. Moreover, a peak voltage of 0.707 was used as the standard, and the continuously frequency that made voltage meet the conditions was defined as the resonance frequency band. In Section 2, the theoretical model of this quarter spherical AEH is discussed, and the relationship of the output voltage with the input sound pressure is shown. In Section 3, the output performance of this quarter spherical AEH, including sound pressure level, output voltage, and output power, are analyzed. Finally, some conclusions are drawn in Section 4.

## 2. Principle and Theory

**Figure 1:**
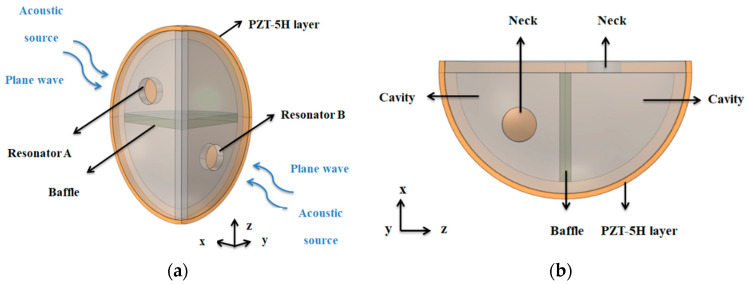
Structure of this quarter spherical acoustic energy harvester (AEH): (**a**) close-up view and (**b**) view in the y-direction.

This quarter-spherical AEH with dual Helmholtz resonators was designed, and two cavities were split equally by a baffle, as shown in Figure 1. The opening directions of the two resonators were set to the x- and y-directions. The two resonators were attached to the same piezoelectric (PZT-5H) layer so that the whole structure could harvest acoustic energy from different directions. This system consisted of two quarter-spherical Helmholtz resonators. However, the opening directions of their necks were different, so that could achieve multi-directional energy harvesting. When excited by incident acoustic wave, the air in the neck moved downward from the static equilibrium position into the cavity. After that, the air in the cavity was compressed. Additionally, the incident sound pressure was amplified by resonator structure so that the piezoelectric film was positively strained under the effect of the intra-cavity pressure. Similarly, when the air in neck area moved down to the extreme position, the internal pressure pushed it back to the upward direction [21]. Due to inertia, the air in neck area returned to the equilibrium position and continued to move upward, and the pressure in cavity was lowered. As a result, the film produced a reverse strain. In this periodic oscillation, an equal amount of positive and negative bound charges appeared on the upper and lower surface of the piezoelectric film. Thus, an effective output voltage was produced.

According to the characteristics of the resonator, when it was excited by external acoustic, both the acoustic resonance and its own mechanical resonance were generated [22]. Therefore, when the two resonant systems were coupled, greater energy could be harvested. This resonator structure could be simplified to a mass-spring system [23]. The air in the neck was equivalent to the mass, and the air in the cavity was equivalent to the spring. The length of acoustic wave was much longer than the radius of the neck. Additionally, the volume of the cavity was much larger than that of the neck. An electro-mechanical-acoustic analogy was used to analyze this quarter spherical AEH. The air motion equation of the neck could be expressed as:

(1) $$M_{m}\frac{dv}{dt}+R_{m}v+\frac{1}{C_{m}}\int vdt=S_{n}p_{i}$$

where *M_m_* is the mass, *v* is the velocity, *R_m_* is the resistance, *C_m_* is the mechanical compliance, *S_n_* is the cross-sectional area of the neck, and *p_i_* is the incident sound pressure.

According to the analogy, the acoustic motion equation can be expressed as [24]:

(2) $$M_{a}\frac{dU}{dt}+R_{a}U+\frac{1}{C_{a}}\int Udt=p_{i}$$

where *M_a_* is the acoustical mass, *U* is the body velocity, *R_a_* is the acoustical resistance, and *C_a_* is the acoustical compliance.

They are expressed, respectively, as

(3) $$U=vS_{n}$$

(4) $$M_{a}=M_{m}/S_{n}^{2}=\frac{\rho L_{eff}}{S_{n}}$$

(5) $$R_{a}=R_{m}/S_{n}^{2}=\frac{\sqrt{32\omega \mu }}{2r_{n}cS_{n}}$$

(6) $$C_{a}=C_{m}S_{n}^{2}=\frac{V_{c}}{\rho c^{2}}$$

where *ρ* is the air density, which is 1.21 kg/m^3^; *L_eff_* is the effective length of neck; ω is the angular frequency of acoustic wave; *μ* is the kinematic viscosity coefficient in air, which is 1.56 × 10^−5^ m^2^/s; *c* is the speed of sound in air, which is 340 m/s; and *V_c_* is the volume of the cavity.

The effective length of neck can be obtained as:

(7) $$L_{eff}=l_{n}+1.7r_{n}$$

where *l_n_* and *r_n_* are the length and radius of the neck, respectively.

The body velocity can be expressed by acoustic impedance *Z* as:

(8) $$U=\frac{p_{i}}{Z}$$

(9) $$Z=R_{a}+j\omega M_{a}+\frac{1}{j\omega C_{a}}$$

The important parameter *G* represents the pressure amplification factor, and it can be obtained as [25]:

(10) $$G=\frac{p_{c}}{p_{i}}=\frac{\left|\frac{1}{j\omega C_{a}}\right|}{\left|R_{a}+j\omega M_{a}-j\frac{1}{\omega C_{a}}\right|}$$

where *p_c_* is the amplified sound pressure.

The highest sound pressure of the cavity was generated at the resonant frequency. The resonant frequency was determined by the cavity volume and the neck dimensions of the resonator [26]. The acoustic resonant frequency and the maximum sound pressure amplification factor are given, respectively, as [27]:

(11) $$f_{a}=\frac{c}{2\pi }\sqrt{\frac{S_{n}}{V_{c}L_{eff}}}$$

(12) $$G_{\max}=2\pi \sqrt{V_{c}{\left(\frac{L_{eff}}{S_{n}}\right)}^{3}}$$

The force *F*_0_ on the piezoelectric layer can be obtained as

(13) $$F_{0}=Gp_{i}S_{p}$$

where *S_p_* is the surface area of the piezoelectric layer.

**Figure 2:**
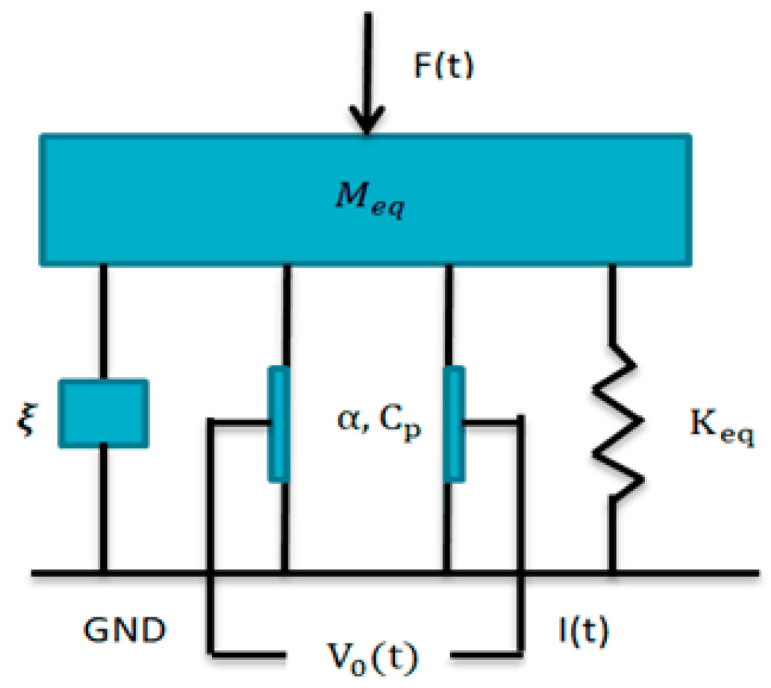
Equivalent model of piezoelectric conversion. *M_eq_* is the equivalent mass, *K_eq_* is the equivalent stiffness, *ξ* is the damping coefficient, *α* is the electromechanical coupling constant, *C_p_* is the equivalent parasitic capacitance, *E* is the elastic modulus, *I* is the moment of inertia, *R_c_* is the outer radius of quarter sphere, and *R_L_* is the load resistance.

An equivalent model to the piezoelectric conversion is shown in Figure 2.

Its equation of motion is shown as [28,29]:

(14) $$M_{eq}\overset{..}{u}(t)+\xi \dot{u}(t)+K_{eq}u(t)-\alpha V_{0}(t)=F(t)$$

The power of piezoelectric layer could be equivalent to the current source. The current in the equivalent circuit could be obtained according to Kirchhoff’s law [30]:

(15) $$\alpha u(t)-C_{p}V_{0}(t)=V_{0}(t)/R_{L}$$

(16) $$K_{eq}=EI/R_{c}^{3}$$

In the lumped parameter model, the vibration displacement could be simply equivalent to a periodic sine wave:

(17) $$u(t)=u_{0}\sin(\omega t-\theta )$$

The excitation of this resonator was in a sinusoidal form. The vibration and the voltage were also in a sinusoidal form, and the period was T. When taking the half period of the vibration (valley ‘a’ to peak ‘b’), Equation (15) can be expressed as:

(18) $$2\alpha u_{0}-2C_{p}V_{0}=\frac{0.707TV_{0}}{2R_{L}}$$

Therefore, the peak voltage is obtained as:

(19) $$V_{0}=\frac{2\omega \alpha R_{L}u_{0}}{2\omega C_{p}R_{L}+0.707\pi }$$

According to Equations (14) and (15), the force can be obtained as:

(20) $$F_{0}^{2}={\left(\left(K_{eq}-M_{eq}\omega^{2}+\frac{\alpha^{2}}{C_{p}}\right)u_{0}-\frac{0.707\pi \alpha }{2\omega C_{p}R_{L}}V_{0}\right)}^{2}+{\left(\xi \omega u_{0}+\frac{V_{0}{}^{2}}{\omega R_{L}u_{0}}\right)}^{2}$$

According to Equations (19) and (20), the displacement *u*_0_ and the peak voltage *V*_0_ can be expressed, respectively, as:

(21) $$u_{0}=\frac{F_{0}}{\sqrt{{\left(K_{eq}-M_{eq}\omega^{2}+\frac{2\omega \alpha^{2}R_{L}}{2\omega C_{p}R_{L}+0.707\pi }\right)}^{2}+{\left(\xi \omega +\frac{4\omega \alpha^{2}R_{L}}{{\left(2\omega C_{p}R_{L}+0.707\pi \right)}^{2}}\right)}^{2}}}$$

(22) $$V_{0}=\frac{2r\Omega }{\left(2r\Omega +0.707\pi \right)\alpha }\times \frac{F_{0}k_{e}^{2}}{\sqrt{{\left(1-\Omega^{2}+\frac{2rk_{e}^{2}}{2r\Omega +0.707\pi }\right)}^{2}+{\left(2\eta +\frac{4rk_{e}^{2}}{{\left(2r\Omega +0.707\pi \right)}^{2}}\right)}^{2}}\Omega^{2}}$$

where $\omega_{n}=\sqrt{\frac{K_{eq}}{M_{eq}}}$ is the natural angular frequency, $k_{e}^{2}=\frac{\alpha^{2}}{K_{eq}C_{p}}$ is the standardized electromechanical coupling coefficient, $\eta =\frac{\xi }{2\sqrt{K_{eq}C_{p}}}$ is the standardized damping ratio, $\Omega =\frac{\omega }{\omega_{n}}$ is the standardized angular frequency, and $r=\omega_{n}C_{p}R_{L}$ is the standardized resistance [30].

## 3. Results and Discussions

The resonator was made of copper, and the piezoelectric layer was made of PZT-5H. The material parameters and structural dimensions of this quarter-spherical AEH are shown in Table 1 and Table 2, respectively.

At the resonant frequency, the relationship of the peak voltage, the neck radius, and the neck length can be expressed as:

(23) $$V_{0}=\frac{2r}{\left(2r+0.707\pi \right)\alpha }\times \frac{p_{i}S_{p}k_{e}^{2}}{\sqrt{{\left(\frac{2rk_{e}^{2}}{2r+0.707\pi }\right)}^{2}+{\left(2\eta +\frac{4rk_{e}^{2}}{{\left(2r+0.707\pi \right)}^{2}}\right)}^{2}}}2\pi \sqrt{V_{c}{\left(\frac{l_{n}+1.7r_{n}}{\pi r_{n}{}^{2}}\right)}^{3}}$$

**Figure 3:**
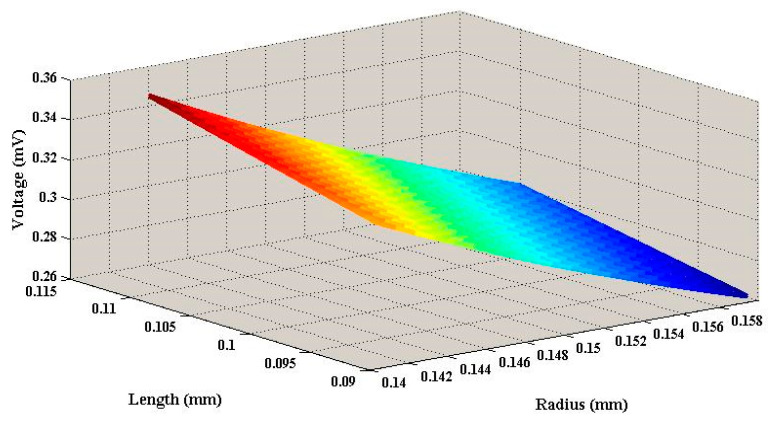
The relationship of the peak voltage with neck radius and length.

As shown in the Figure 3, when the neck length was 0.1 mm, the neck radius was the independent variable and ranged from 0.14 to 0.16 mm. The peak voltage decreased from 0.344 to 0.270 mV. At the same time, when the neck radius was 0.15 mm, the neck radius was the independent variable and ranged from 0.09 to 0.11 mm. The peak voltage increased from 0.294 to 0.318 mV. The size of the neck and cavity determined the voltage by affecting the amplification of the sound pressure, which can be seen from Equations (7) and (12). The voltage was proportional to neck length and cavity volume, and it was inversely proportional to neck radius. Because the cavity volume was inversely proportional to baffle thickness, the voltage was inversely proportional to baffle thickness.

Since the increase of PZT thickness generated more charge, the output voltage was proportional to PZT thickness. Moreover, the increase of bottom thickness caused an addition to the moment of inertia, so it was inversely proportional to output voltage.

However, when the size of an AEH is chosen, the coupling of the two resonance frequencies must be considered. The highest energy collection efficiency can be obtained when the mechanical resonance and the resonance of sound pressure are the same. By adjusting the size of the resonator’s neck, the resonant frequency of sound pressure is changed to make it close to the mechanical resonant frequency. Here, when the radius of the neck was 0.15 mm and the length of the neck was 0.1 mm, the mechanical resonant frequency (fn) was about 41 kHz. According to Equation (11), the acoustic resonant frequency was about 41.04 kHz.

This AEH was simulated with Comsol 5.3 a, and the solid element was a three-dimensional hexahedron. The physical fields used in the analysis included structural mechanics, pressure acoustics, static electricity, and electrical circuits. The front, bottom, and left sides of the cube were set as the incident pressure field. Additionally, the back, top, and right sides of the cube were set as hard sound field boundaries (no reflection wave). The acoustic source was set as a far-field plane wave with a 110 pa acoustic pressure. The direction of transmission was e = (−1, −1, 1). In an ideal medium, sound pressure amplitude does not change with the distance and there is no energy loss during transmission. Moreover, its wavelength is far longer than the length of neck, so it meets the conditions of the air motion equation in resonators. Here, the AEH was set in the middle of a cube with a 4 mm side length and the air was used as the medium. The piezoelectric film was in the boundary condition of mechanical clamping and electrical short circuit. The positive and negative electrodes were, respectively, set on the upper and lower surfaces of the piezoelectric film to connect with external resistors. The device was periodically strained by the amplified incident sound wave, and the strain was converted into voltage output due to the piezoelectric effect. In the end, the grid was checked from regular to fine, and the minimum unit was 0.02 mm. The result was basically unchanged.

### 3.1. Input Sound Pressure Level

According to Equation (12), the largest magnification for the external sound pressure through the cavity was about 43. The sound pressure level can be expressed as [31]:

(24) $$SPL=20\mathrm{lg}\frac{p}{p_{ref}}$$

where *P_ref_* is reference sound pressure (2 × 10^−5^ pa).

**Figure 4:**
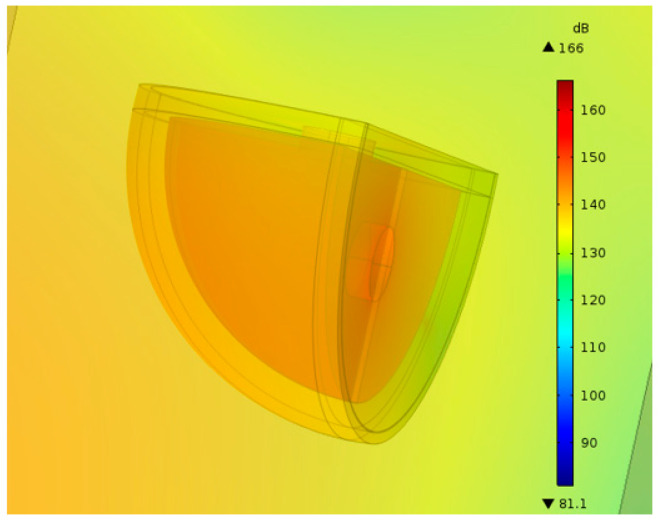
The simulation results of sound pressure level.

When the external sound pressure was 110 pa, the equivalent sound pressure level was about 135 dB. The sound pressure was amplified by 43 times, which was equal to the sound pressure level increased by about 32 dB. In the theorical result, the sound pressure level in the cavity was about 167 dB. As shown in Figure 4, the largest simulated sound pressure level was 166 dB at 41 kHz. It was found that the theorical and simulated results were almost the same, and the dual-resonator structure played a role in sound pressure amplification.

### 3.2. Output Voltage

**Figure 5:**
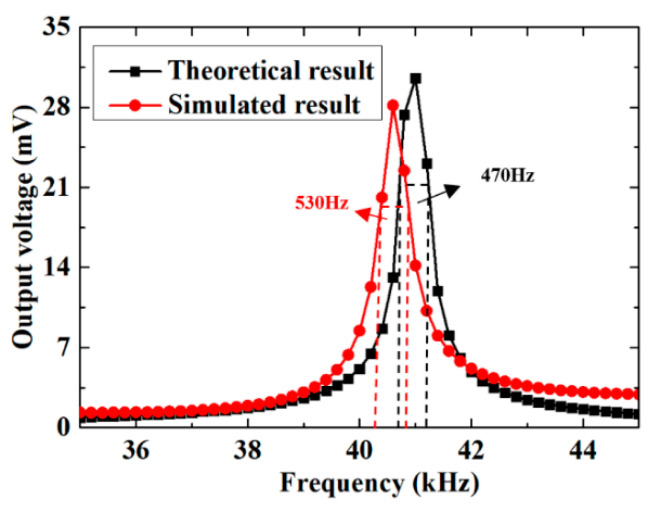
Comparison of theoretical and simulated results.

In order to obtain the output voltage of this quarter-spherical AEH, the incident acoustic frequency was set as an independent variable, ranging from 35 to 45 kHz with an interval of 0.2 kHz. As shown in Figure 5, the theoretical result showed that the maximum output voltage was about 30 mV at 41 kHz. The simulated result showed that the maximum output voltage was about 28 mV at 40.6 kHz. The theoretical result of frequency band was 470 Hz, and the simulated result was 530 Hz. The difference between theoretical results and simulation results was as follows: on the one hand, it was possible that an equivalent mass deviation existed in $\omega_{n}=\sqrt{\frac{K_{eq}}{M_{eq}}}$. On the other hand, Equation (22) did not calculate the stress between the shear oscillator arrays. Compared with the related structures, as shown in Table 3, the frequency band of this quarter spherical acoustic energy harvester was wider.

Since the size of the neck was slightly smaller than the wavelength of the acoustic wave, the diffraction of the acoustic wave was caused, thus making the acoustic waves propagate in different directions after entering the cavity and not affecting the sound wave collection before entering the cavity. Acoustic waves are elastic mechanical vibration waves that have strong directivity in ideal air media. The sound source in the simulation propagated directionally, which is called a wave beam. A resonator can only absorb sound waves in the opening direction of its neck. Therefore, this AEH with dual-directional resonators could double the sound wave utilization rate.

**Figure 6:**
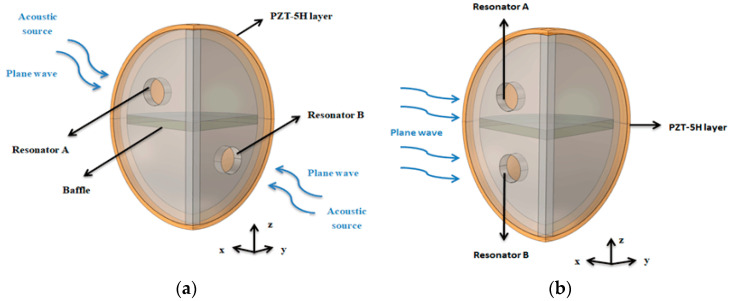
Schematic diagram of (**a**) unidirectional coupled resonators and (**b**) dual-directional coupled resonators.

**Figure 7:**
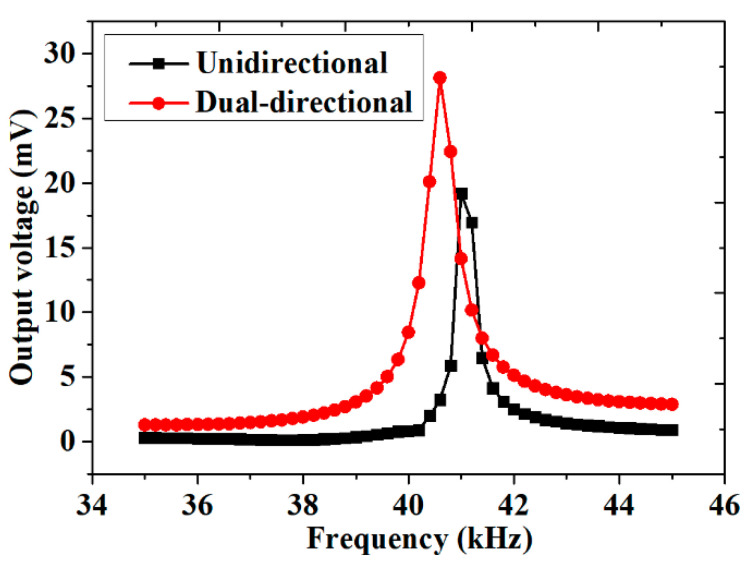
The output voltage for unidirectional and dual-directional coupled resonators.

In order to verify the effectiveness of the necks in different directions, the AEH with two unidirectional resonators and the AEH with dual-directional resonators were studied, as shown in Figure 6. As shown in Figure 7, the maximum output voltage of the AEH with unidirectional resonators was about 19.2 mV at 41 kHz. The maximum output voltage of the AEH with dual-directional resonators was about 28 mV at 40.6 kHz. The output voltage of the AEH with unidirectional resonators was only 68.6% of that of this AEH with dual-directional resonators. It could be seen that the collection efficiency of this AEH with dual-directional resonators was higher.

In the previous design, the size of the two resonators was the same to make their acoustic resonant frequency consistent with the mechanical resonant frequency. When one of the acoustic resonant frequencies was changed a little, it produced a secondary output peak, thus achieving the purpose of broadening the resonant frequency band. According to Equation (11), the acoustic resonant frequency was inversely proportional to the length of neck.

**Figure 8:**
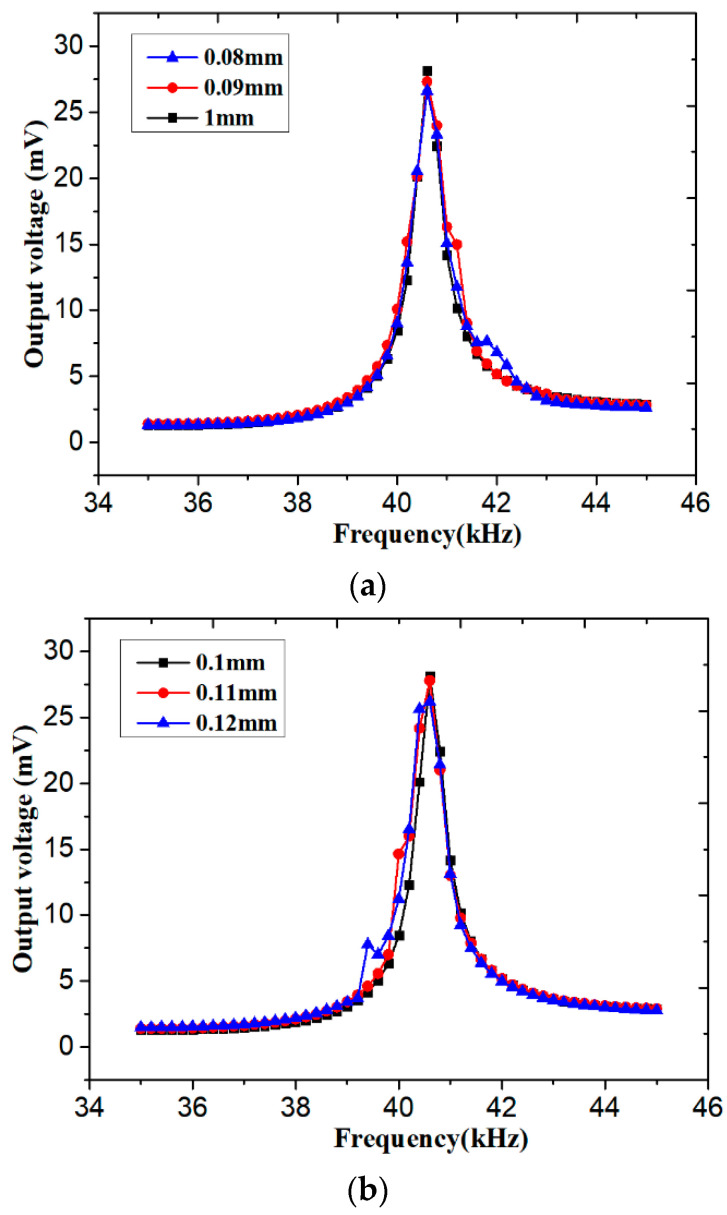
The output voltage with different ln2 values: (**a**) less than 0.1 and (**b**) more than 0.1.

We kept the neck length of one resonator (ln1 = 0.1 mm) unchanged, and then we adjusted the neck length of the other resonator (ln2). As shown in Figure 8a, when ln2 was 0.08 mm, the secondary output voltage was about 7.6 mV at 41.8 kHz. Taking 7 mV as a standard effective voltage, the effective frequency band increased by about 28.5%. When ln2 was 0.09 mm, the secondary output voltage was about 15 mV at 41.2 kHz. When taking 15 mV as a standard effective voltage, its effective frequency band increased about 43%. In both two cases, the left part of the output curve was almost same as the previous one, and the resonant frequency band was widened to the right.

As shown in Figure 8b, when ln2 was 0.11 mm, the secondary output voltage was about 15 mV at 40 kHz. Its effective frequency band increased by about 42%. When ln2 was 0.12 mm, the secondary output voltage was about 8 mV at 39.4 kHz. The effective frequency band increased by about 31%. In both two cases, the right part of the output curve was almost same as the previous one, and the resonant frequency band was widened to the left.

Moreover, the more the secondary peak offset, the lower the maximum output voltage was. Therefore, it is necessary to pay attention to achieving a balance between high output voltage and a wide frequency band.

### 3.3. Output Power

Output power can be expressed as [35]:

(25) P0=Vrms2R=V0/222R=V028R

where *V_rms_* is the root mean squared voltage, *V*_0_ is the peak voltage, and *R* is the external resistance.

The input power is produced by the incident sound wave, which can be expressed as:

(26) $$P_{i}=\frac{p_{i}^{2}S_{n}}{\rho c}$$

where *p_i_* is the sound pressure, *S_n_* is the opening area, *ρ* is the air density, and *c* is the sound speed.

The energy conversion efficiency is:

(27) $$\eta =\frac{P_{0}}{P_{i}}$$

**Figure 9:**
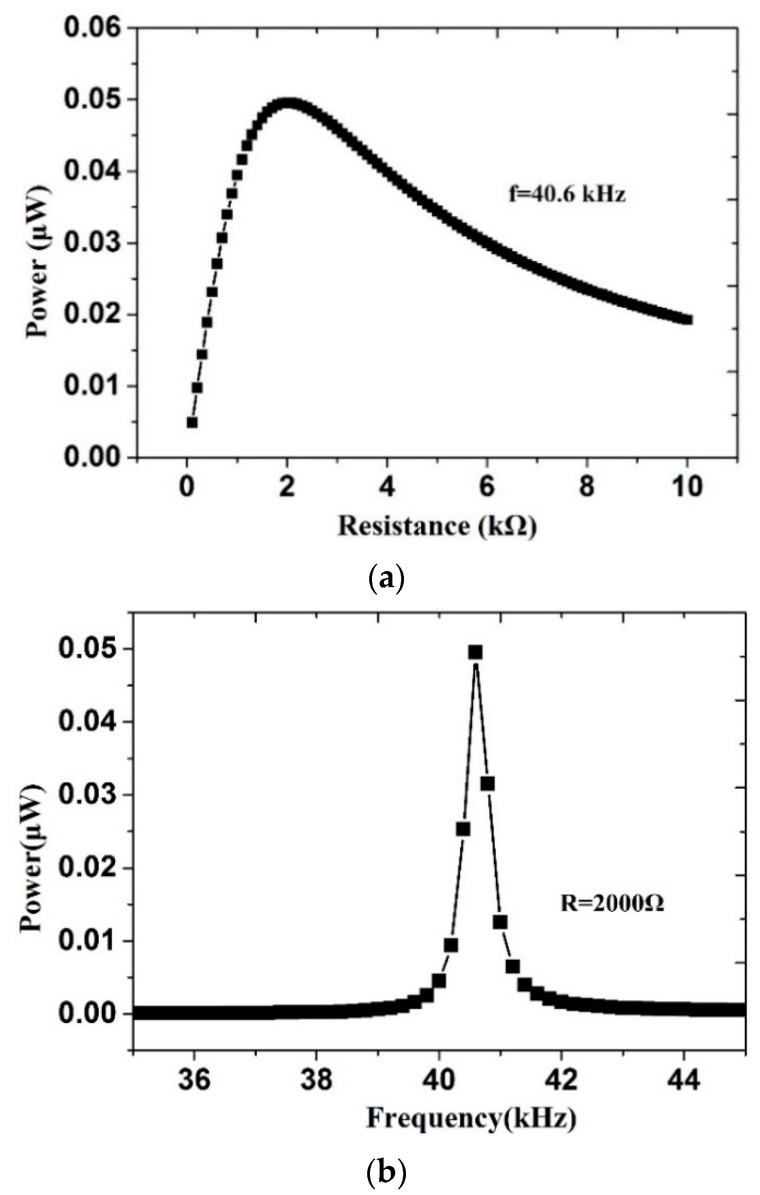
The relationship of output power: (**a**) with external resistance and (**b**) with frequency.

Here, the input power could be calculated as 1.95 μW. Output power could be obtained when the external resistance was equal to the equivalent impedance of system. The resistance *R* was set as an independent variable. The resistance was changed from 0.1 to 10 kΩ. As shown in Figure 9, when the external resistance was 2 kΩ and the resonant frequency was 40.6 kHz, the highest output power was about 0.05 μW. The efficiency of dual-directional energy harvesting was 2.56%.

Based on the above information, it was found that considerable output power could be obtained with the dual-resonator structure. As shown in Table 4, with a similar acoustic pressure level, the power density of this quarter-spherical AEH was higher than other piezoelectric energy harvesters. In addition, this structure had the advantage of collecting multi-directional energy, so it could improve the energy conversion efficiency of acoustic sources in the environment.

## 4. Conclusions

In summary, a quarter-spherical acoustic energy harvester was designed with dual Helmholtz resonators. This AEH can harvest acoustic energy in dual directions. Moreover, the resonant frequency band can be broadened by adjusting the structural dimension. According to the theoretical and simulated results, this structure has considerable output performance. Its frequency band and power density were about 470 Hz and 12.7 µWcm^−2^, respectively, which were both improved compared to a baseline device. In the future, this structure has broad prospects in implantable medical devices.

## Figures and Tables

**Table 1:** Material parameters.

	Young’s Modulus (GPa)	Relative Dielectric Constant	Piezoelectric Constant (C/m^2^)	Mass Density (Kg/m^3^)
Cu	110			8960
PZT-5H	56	1433.6	−6.62	7500

**Table 2:** Structural dimensions.

Radius of Cavity (Mm)	Length of Neck (Mm)	Radius of Neck (Mm)	Thickness of Bottom (Mm)	Thickness of PZT (Mm)	Thickness of Baffle (Mm)
*R_c_*	*l_n_*	*r_n_*	*t*_1_	*t*_2_	*t*_3_
1	0.1	0.15	0.1	0.05	0.1

**Table 3:** Comparisons of working frequency band.

Reference	Material	Frequency Band
[32]	PZT-5H	Less than 10 Hz
[33]	PZT Pb(Zr_1−x_Ti_x_)O_3_	Less than 50 Hz
[12]	PZT	Less than 60 Hz
[34]	PZT-5H	300 Hz
This work	PZT-5H	470 Hz

**Table 4:** Comparison of acoustic energy harvesters based on resonator structures.

Author (Reference Number)	Energy Source	Type	Material	Dimensions	Resonant Frequency (kHz)	Power Density (µW cm^−2^)
Horowitz et al. [21]	Acoustic pressure (149 dB)	Diaphragm (ring)	PZT	d1 = 2.4 mm d2 = 2.23 mm hp = 0.267 µm	13.6	0.34
Kimura et al. [36]	Acoustic pressure (100 dB)	Diaphragm	PZT	d = 1.2 mm hp = 1.0 µm	16.7	0.0098
Li et al. [37]	Acoustic pressure (100 dB)	Cantilever	PZT	L = 40 mm b = 20 mm hp = 0.48 mm	0.199	57.4
This work	Acoustic pressure (135 dB)	Diaphragm	PZT	d = 1.1 mm hp = 0.05 mm	40.6	12.7
